# Optimization of Extraction, Chemical Profiling, and Evaluation of Antioxidant and Antibacterial Activities of Cortex Dictamni Extract

**DOI:** 10.3390/molecules31111869

**Published:** 2026-05-29

**Authors:** Hui Chen, Mingfang Teng, Hang Zhao, Huining Zhang, Meihui Yang, Peng Zhang, Rongfeng Chen

**Affiliations:** 1 National Medical Products Administration Institute of Executive Development, Beijing 100073, China; 2 College of Life Engineering, Shenyang Institute of Technology, Fushun 113122, China; 3 National Center for Occupational Safety and Health, National Health Commission, Beijing 102308, China

**Keywords:** Cortex Dictamni, alkaloids, extraction process, antibacterial, antioxidant, chemical composition

## Abstract

Cortex Dictamni, the dried root bark of the plant *Dictamnus dasycarpus Turcz*. from the Rutaceae family, contains alkaloids as its primary bioactive components, which exhibit a wide range of pharmacological effects. This study aims to optimize the extraction process and to characterize the chemical composition of Cortex Dictamni extract (CDE), as well as to evaluate its in vitro antioxidant and antibacterial activities. The response surface methodology (RSM) was employed to optimize the extraction process of CDE, while Q-Orbitrap high-resolution liquid chromatography–mass spectrometry (LC-MS) was utilized to identify the chemical components in the CDE. Antioxidant and antibacterial activities were evaluated using ABTS and DPPH radical scavenging assays and by determining the minimum inhibitory concentration (MIC), respectively. The results indicated that the optimized extraction yield of CDE was 44.73%. The optimal extraction conditions were: ethanol concentration of 70%, material-to-liquid ratio of 1:40 g/mL, and extraction time of 35 min. A total of 10 components were identified in the extract, including alkaloids, lactones, terpenes, and flavonoids. The extract demonstrated notable oxygen-free radical scavenging capabilities, as evidenced by its DPPH (IC50 = 0.316 mg/mL) and ABTS (IC50 = 0.166 mg/mL) values. Additionally, it exhibited antibacterial effects, particularly against Escherichia coli and Pseudomonas aeruginosa, with a minimum inhibitory concentration (MIC) of ≥128 mg/mL. CDE may serve as a potential natural compound for applications in the food and pharmaceutical industries.

## 1. Introduction

*Dictamnus dasycarpus Turcz*. is widely distributed in China, Korea, Mongolia, and Russia [1]. Cortex Dictamni is the dried root bark of *Dictamnus dasycarpus Turcz*. and is traditionally used in Chinese medicine for clearing heat and drying dampness, dispelling wind, and relieving itching, as well as detoxifying the body [2]. Its main active components include alkaloids, limonoids, lactones, sesquiterpenes, and their glycosides [3]. With advances in modern pharmacological research, the biological activities of Cortex Dictamni have been increasingly reported, particularly its antibacterial, anti-inflammatory, and anti-allergic effects [4,5,6], attracting increasing attention from researchers worldwide.

Since the 1980s, progress has been made in identifying active components from Cortex Dictamni extracts (CDEs). In the early stages, techniques such as thin-layer chromatography (TLC), gas chromatography (GC), and high-performance liquid chromatography (HPLC) were employed to preliminarily identify major constituents, including alkaloids, limonoids, lactones, sesquiterpenes, and their glycosides [7,8]. In the 21st century, high-resolution techniques such as mass spectrometry (MS) and nuclear magnetic resonance (NMR) have enabled more in-depth studies of the quantitative analysis and structural elucidation of active compounds. Advanced techniques, including LC–MS and tandem mass spectrometry (MS/MS), have enabled the structural identification of limonoid compounds in Cortex Dictamni, including phellodendrin-13-O-β-D-glucopyranoside. NMR analysis has been utilized to precisely determine the structure of Dictamnine, confirming it as 11,13-dihydroxy-9,13-dimethoxybiphenyl-3,4-dione [9,10,11].

Microbial infections and reactive oxygen species (ROS) remain key concerns in public health. Natural compounds derived from plants exhibit a wide range of biological activities, primarily including antibacterial and antioxidant effects. CDEs demonstrate significant in vitro free radical scavenging capacity. This activity is largely attributed to their high content of phenolic and flavonoid compounds. Rutin, quercetin, naringenin, diosgenin, and oleandrin are reported in Cortex Dictamni [12,13]. The hydroxyl group at the C3 position of the C-ring and the hydroxyl groups at the 3′ and 4′ positions of the B-ring in flavonoids such as rutin and quercetin are key functional groups for scavenging hydroxyl radicals (HO•). In antibacterial studies, CDEs and their monomeric compounds—particularly dictamnine, obacunone, and fraxinellone—exhibit notable inhibitory effects against multiple bacterial strains [14,15]. Therefore, products prepared from CDEs may serve as potential natural antioxidants and antibacterial agents with promising market development potential.

Although Cortex Dictamni contains diverse bioactive constituents, alkaloids are considered one of the most representative and abundant components with reported antimicrobial and antioxidant properties. Therefore, the extraction yield of total alkaloids was used as the response variable for process optimization. However, given that other compounds may also contribute to the overall bioactivity, the chemical profile of the crude extract was comprehensively analyzed, and both antioxidant and antibacterial activities were evaluated without attributing the effects solely to alkaloids. Nevertheless, systematic optimization of the extraction process using response surface methodology (RSM), comprehensive chemical profiling via high-resolution LC-MS, and simultaneous evaluation of both antioxidant and antibacterial activities have not been previously reported for Cortex Dictamni. In this study, ultrasonic-assisted extraction was employed to optimize the isolation of total alkaloids from Cortex Dictamni. Chemical profiling and evaluation of in vitro antioxidant and antibacterial activities were then performed, providing a reference for further development and application.

## 2. Results and Discussion

### 2.1. Validation of the Bromocresol Green Spectrophotometric Method for Total Alkaloid Quantification

The absorbance values of five replicate measurements were 0.025, 0.025, 0.025, 0.024, and 0.025. The relative standard deviation (RSD) was 1.50%, which is <2.00%, indicating good precision of the bromocresol green spectrophotometric method. The stability of the test sample was evaluated within 1.5 hours, and the absorbance RSD was 1.90% (<2.00%), demonstrating that the sample remained stable during this period. The repeatability of the method was confirmed by measuring five replicate samples, yielding absorbance values of 0.029, 0.029, 0.030, 0.029, and 0.029, with an RSD of 1.53% (<2.00%). These results indicate that the bromocresol green spectrophotometric method is suitable for the determination of active component contents in Cortex Dictamni and for quantitative analysis in subsequent response surface methodology (RSM) experiments. In addition, we have explicitly stated that total alkaloid content is expressed as Dictamnine equivalents.

### 2.2. Results of the Single-Factor Experiments

**Figure 1:**
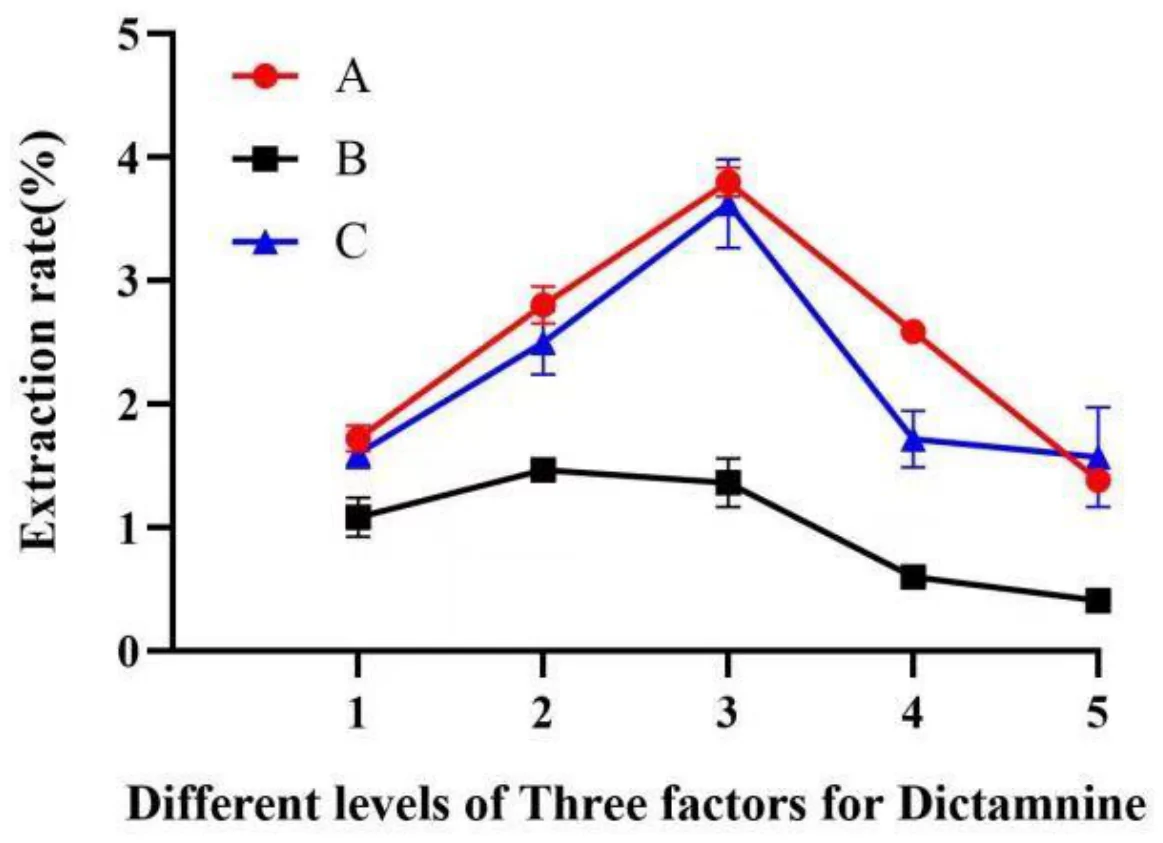
Effects of material-to-liquid ratio (A, mL/g), ethanol concentration (B, %), and extraction duration (C, min) on the extraction efficiency of CDE.

As shown in Figure 1, increasing the material-to-liquid ratio led to a corresponding increase in extraction yield, which peaked at 1:40 g/mL. However, further increases in solvent volume resulted in a decrease in extraction yield. The extraction yield gradually increased when the ethanol concentration ranged from 55% to 65%, but decreased as the concentration exceeded this range. Additionally, the extraction yield increased continuously during 20–40 min of extraction but decreased when the extraction time was extended to 40–60 min.

### 2.3. Results of the Optimization of Extraction Conditions by Box–Behnken Design

As shown in Table 1, a total of 17 experiments were conducted, with the extraction yield selected as the response variable to optimize the extraction process of alkaloids from Cortex Dictamni. Multivariate regression analysis was performed using Design Expert software (version 8.0.6) to statistically fit the final equation of extraction yield. Based on the regression analysis and integration of the experimental data, the obtained quadratic multiple regression equation was: Y = 2.63−0.0063 A−0.0663 B + 0.0725 C + 0.1200 AB + 0.0425 AC−0.0775 BC−0.6980 A2−0.6680 B2−0.8555 C2.

As shown in Table 2, within the range of the experiment, the model was highly significant (*p* = 0.0011 < 0.0100), indicating a clear linear relationship between the model variables and the response values. The lack-of-fit term was not significant (*p* = 0.4298 > 0.05), suggesting minimal influence of non-experimental factors on the total alkaloid extraction yield. The close agreement between experimental and predicted values demonstrates high accuracy of the data. The regression coefficient (R^2^ = 0.9477) further confirms the good fit and high reliability of the model.

**Figure 2:**
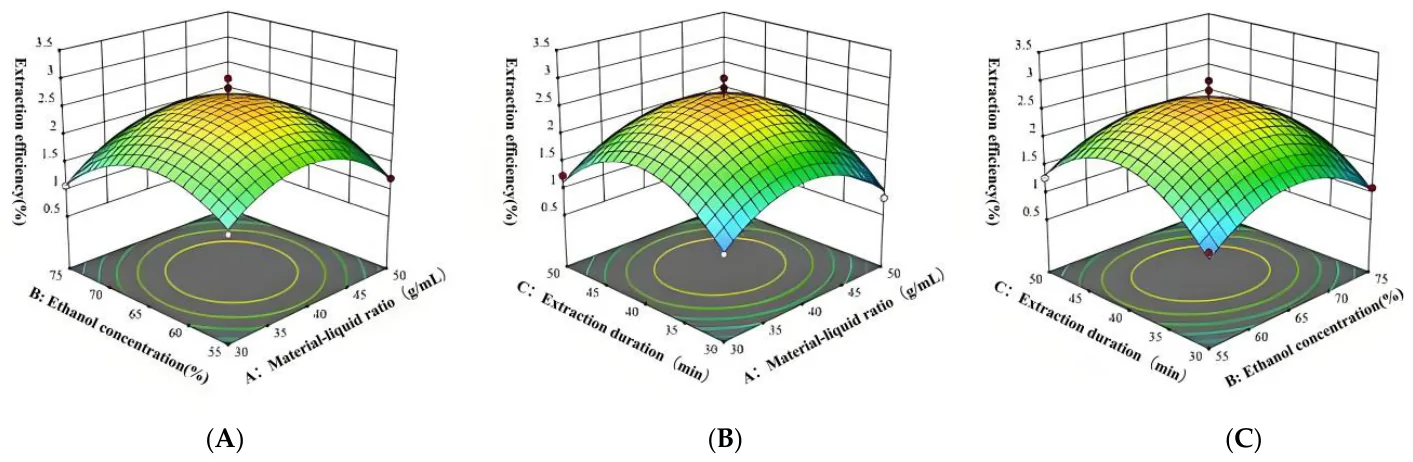
Interaction effects of (**A**) material-to-liquid ratio and ethanol concentration, (**B**) material-to-liquid ratio and extraction duration, and (**C**) ethanol concentration and extraction time on the extraction efficiency of Dictamnine.

Based on the analysis performed using Design-Expert 13.0 software, the response surface was generated. As shown in Figure 2A, when extraction time is fixed at the zero level and the ethanol concentration is constant, the total alkaloid extraction yield initially increases and then decreases with an increasing material-to-liquid ratio. Conversely, when the material-to-liquid ratio is fixed, the extraction yield also exhibits a similar trend with rising ethanol concentrations. Figure 2B indicates that with the ethanol concentration at the zero level, the extraction yield first rises and then falls as the material-to-liquid ratio increases. Increasing the extraction time further accentuates this trend, suggesting that extraction time exerts a greater influence on the extraction yield than ethanol concentration. Figure 2C shows that when the material-to-liquid ratio is fixed at the zero level and the ethanol concentration is constant, the extraction yield increases initially and then decreases with a longer extraction time. At a fixed extraction time, the yield also follows a similar pattern with increasing ethanol concentrations, though the effect is less pronounced than that of extraction time.

Ultrasonic extraction technology, utilizing cavitation, mechanical vibration, and thermal effects, effectively disrupts plant cell walls and accelerates the dissolution of intracellular secondary metabolites. This method offers advantages such as a short extraction time, high efficiency, and reduced solvent consumption [16]. Lin Hao et al. compared reflux extraction, ultrasonic extraction, and composite enzymatic hydrolysis for white fresh bark. The results revealed that ultrasonic extraction yielded the highest alkaloid content and exhibited the strongest biological activity [17]. Based on the regression equation obtained from the software, the optimal extraction conditions for Cortex Dictamni crude extract were determined as follows: material-to-liquid ratio of 1:41.42 g/mL, ethanol concentration of 69.13%, and extraction time of 34.13 min. For practical considerations in reagent production and application, the extraction parameters were adjusted to a material-to-liquid ratio of 1:40 g/mL, ethanol concentration of 70%, and extraction time of 35 min. Under these conditions, the predicted extraction yield of GRS is 43.14%.

### 2.4. Identification of Chemical Components in CDE

LC–MS was used to characterize the chemical components of the extract, which were then compared with several databases, iincluding mzCloud, mzVault, ChemSpider, and a secondary mass spectrometry database for Chinese herbal medicines independently established by Sanshu Biology. For representativeness, compounds with peak areas exceeding 1 × 10^8^ were selected for further analysis. The retention times (R), [M − H]^−^, MS/MS [M − H]^−^, [M + H]^+^, MS/MS [M + H]^+^, calculated masses, and molecular formulas of each component are listed in Table 3. Fragment ions of compounds **1** and **2** were compared with previously reported data [18,19]; the ions at *m*/*z* 200.1 and 169.1 corresponded to Dictamnine and Norharman, respectively. Similarly, fragments observed at *m*/*z* 260.1 and 138.1 likely correspond to Skimmianine [20] and Trigonelline [21]. Ions at *m*/*z* 609.2 and 471.2 are considered characteristic fragments of hesperidin and limonoids (compounds **5** and **6**) [22,23], while ions at *m*/*z* 459.1 and 455.2 corresponded to dictamnine glycoside (compound 7) [24] and obacunone (compound **8**) [25], respectively. The ions at *m*/*z* 177.1 and 333.2 belong to sedanolide (compound **9**) [26] and andrographolide (compound **10**) [27]. Of the ten compounds identified, norharman, dictamnine, skimmianine, and trigonelline are alkaloids; limonin and obacunone are terpenoids; sedanolide and andrographolide are lactones; and phlorizin and neohesperidin are flavonoids.

### 2.5. Antioxidant Capacity

1,1-Diphenyl-2-trinitrobenzenehydrazone (DPPH) is a highly stable nitrogen-centered free radical that can be scavenged by nitrogen atom inhibitors, resulting in a decrease in color intensity measured by UV spectrophotometry. Due to its reproducibility, DPPH is widely used to assess antioxidant activity. As shown in Table 4, the ethanol extract of Cortex Dictamni exhibited concentration-dependent DPPH radical scavenging, with an IC_50_ value of 0.316 mg/mL. At 1 mg/mL, the extract achieved a maximum scavenging rate of 82.30%. Similarly, the extract showed enhanced ABTS^+^ radical scavenging capacity with increasing concentrations, presenting a good dose-dependent response and an IC_50_ of 0.166 mg/mL. At 1 mg/mL, the maximum scavenging rate reached 93.5%. Previous studies have also demonstrated that water extracts of Cortex Dictamni exhibit dose-dependent scavenging of DPPH and ABTS radicals and inhibit lipid peroxidation, with an inhibition rate of 40.6 ± 5.2% [28]. Chen et al. reported that the acetone extract of Cortex Dictamni is an effective free radical scavenger with notable antioxidant capacity [29]. In addition to the synergistic effect of the extract as a whole, individual compounds such as apocynin, andrographolide, and neohesperidin have been shown to possess significant antioxidant activity [30,31,32].

### 2.6. Antibacterial Capacity

With the increasing emergence of drug-resistant strains, the antibacterial properties of medicinal plants have received growing attention. Alkaloids exhibit broad-spectrum antibacterial activity through mechanisms such as inhibition of cell wall synthesis and alteration of membrane permeability [33]. As shown in Table 5, the ethanol extract of Cortex Dictamni (CDE) exhibited antibacterial effects against *E. coli* and *P. aeruginosa* with MIC values = 128 mg/mL, followed by *L. monocytogenes* (MIC = 256 mg/mL), but showed no obvious inhibition against *S. aureus*. For comparison, conventional antibiotics such as doxycycline typically display MICs of 0.5 μg/mL against E. coli [34], indicating that CDE is approximately 256,000-fold less potent. However, Chen et al. reported that an aqueous extract of Cortex Dictamni (0.4 g/mL) completely inhibited *S. aureus* and *E. coli* within 20 min [3], consistent with the antibacterial effects observed in our study. Tian also isolated two furoquinoline alkaloids from *Dictamnus dasycarpus Turcz*., exhibiting MIC = 64 μg/mL against *P. aeruginosa* [35], roughly 2000-fold more potent than the crude extract. These findings suggest that the antibacterial activity of CDE may be attributed to individual compounds such as skimmianine, limonoids, and trigonelline [36,37,38], warranting further fractionation and investigation.

## 3. Materials and Methods

### 3.1. Reagents

Chromatograph: UltiMate 3000 RS (Thermo Fisher Scientific, Waltham, MA, USA); Mass spectrometer: Q Exactive high-resolution mass spectrometer (Thermo Fisher Scientific, USA); UV-Vis spectrophotometer: 7230G (Shanghai Youke Instrument Co., Ltd., Shanghai, China). Ultrasonic cleaner (KQ-400DE, Kunshan Shumei, Kunshan, China) Standard substances of rutin (MB5118) and vitamin C (VC, MB4168) were purchased from Dalian Meilun Biotechnology Co., Ltd. (Dalian, China) 2,2′-azino-bis(3-ethylbenzothiazoline-6-sulfonate) and 1,1-diphenyl-2-trinitrophenylhydrazine (DPPH) were purchased from Beijing Solarbio Science & Technology Co., Ltd. (Beijing, China). *Staphylococcus aureus* (BNCC186335), *Escherichia coli* (BNCC133264), *Salmonella* (BNCC358231), *Listeria monocytogenes* (BNCC185986), and *Pseudomonas aeruginosa* (BNCC337005) were purchased from BeNa Culture Collection (Beijing, China).

### 3.2. Samples and Processing

Cortex Dictamni was purchased from Liaoning University of Traditional Chinese Medicine Affiliated Hospital and authenticated by Professor Junfan Fu, a botanist from Yancheng Teachers University. The plant material was cleaned, naturally dried, ground into a fine powder, and passed through a 200-mesh sieve for subsequent experiments.

### 3.3. Determination Method for the Content of Dictamnine in the Extract

#### 3.3.1. Precision Test

An accurate volume of 0.2 mL of the test sample was taken in five replicates. To each sample, 5 mL of buffer solution and 2 mL of bromocresol green indicator solution were added, and the mixture was shaken for 1 min. Subsequently, 10 mL of chloroform was added, followed by an additional 1 min of shaking. After standing for 30 min, the absorbance was measured at 419 nm, and the relative standard deviation (RSD) of the results was calculated.

#### 3.3.2. Stability Test

A volume of 0.2 mL of the test sample was taken, to which 5 mL of buffer solution and 2 mL of bromocresol green indicator solution were added. The mixture was shaken for 1 min, followed by the addition of 10 mL of chloroform and an additional 1 min of shaking. After standing for 30 min, samples were collected every 0.5 hours for a total of three times. Absorbance was measured at 419 nm, and the RSD of the results was calculated.

#### 3.3.3. Repeatability Test

Five replicates of 5.00 g of Cortex Dictamni powder from the same batch were precisely weighed. Test sample solutions were prepared as described in Section 3.3.1. For each solution, 0.2 mL of each solution was taken, to which 5 mL of buffer solution and 2 mL of bromocresol green indicator solution were added. The mixtures were shaken for 1 min, followed by the addition of 10 mL of chloroform and an additional 1 min of shaking. After standing for 30 min, absorbance was measured at 419 nm, and the relative standard deviation (RSD) of the results was calculated.

### 3.4. Preparation of Standard Curve

Referring to the method of Ajanal et al. [39], 3.0 mg of Dictamnine standard was weighed and placed into a 10 mL volumetric flask. The standard was dissolved in 1% acidic ethanol and diluted to the mark, yielding a reference solution with a concentration of 0.327 mg/mL. Aliquots of 0.1, 0.2, 0.4, 0.6, and 0.8 mL of the reference solution were transferred into separate 50 mL centrifuge tubes and diluted to 1 mL with 1% acidic ethanol. Subsequently, 5 mL of buffer solution and 2 mL of bromocresol green indicator solution were added to each tube, followed by shaking for 1 min. 10 mL of chloroform was added and the mixture was shaken for additional 1 min before standing for 30 min. Absorbance was measured at 419 nm. With absorbance (Y) as the vertical coordinate and reference solution concentration (X) as the horizontal coordinate, a standard curve was plotted, resulting in Equation (1):

Y = 0.0346 X + 0.0125 (R^2^ = 0.9930) (1)

### 3.5. Extraction Process of CDE

The ultrasonic extraction method described by Teng et al. [40] was referenced and modified according to the present experimental conditions. Five grams of coarse Cortex Dictamni powder was weighed. Ethanol of varying concentrations was added according to the specified material-to-liquid ratio, and the mixture was soaked for 30 min. Ultrasonic treatment was performed at 400 W for varying durations, with each treatment repeated three times. After ultrasonic extraction, the mixture was filtered under vacuum, and the filtrates were combined. The solvent was evaporated in a water bath until no ammonia odor remained and then concentrated to dryness under reduced pressure to yield the crude CDE. It should be noted that the resulting CDE is a crude ethanol extract, not a purified alkaloid fraction. The content of Dictamnine in the crude extract was calculated using Equation (2):

CDE yield (%) of Cortex Dictamni (%) = (C × V × N)/M × 100%(2)

where C is the Dictamnine content in the crude extract (mg/mL), V is the volume of crude extract (mL), N is the dilution factor, and M is the mass of Cortex Dictamni powder weighed (g).

### 3.6. Single-Factor Experiments

Single-factor experiments were conducted on three main factors—ethanol concentration, ultrasonic time, and liquid-to-material ratio—with the total alkaloid extraction yield of Cortex Dictamni as the evaluation index. The experimental ranges were set as follows: material-to-liquid ratios from 1:20 to 1:60 mL·g^−1^, ethanol concentrations from 55% to 95%, and ultrasonic times from 20 to 60 min. When assessing the effect of a single factor, the other two factors were fixed at their respective third levels.

### 3.7. Optimization of Extraction Conditions by Box–Behnken Design

As shown in Table 6, based on the analysis of the effects of various factor levels on the total alkaloid extraction yield of Cortex Dictamni, three experimental factors were selected as variables: liquid-to-material ratio (A), ethanol concentration (B), and ultrasonic time (C). A three-factor, three-level Box–Behnken response surface experiment was designed using Design-Expert 13.0 software. A regression equation was fitted to optimize the extraction process of CDE.

### 3.8. Detection of Dictamnine Chemical Components in Cortex Dictamni

#### 3.8.1. Sample Preparation

Referring to the method of Chang et al. [41], approximately 100 mg of Cortex Dictamni powder was weighed and mixed with 1 mL of 80% methanol by vortexing. Two to three zirconia grinding beads were added, and the sample was subjected to grinding for 5 min, followed by vortexing for 10 min. The mixture was then centrifuged at 13,000 rpm and 4 °C for 10 min. The supernatant was filtered through a 0.22 μm membrane filter, and the filtrate was collected for subsequent instrumental analysis.

#### 3.8.2. Detection Conditions

Mass spectrometry conditions: The ion source was electrospray ionization (ESI), and the scan mode was set to polarity switching between positive and negative ions. Detection was performed in Full mass/dd-MS^2^ mode, with a resolution of 70,000 for full MS and 17,500 for dd-MS². The scan range was 100.0–1500.0 *m*/*z*. The spray voltage was 3.2 kV (positive mode), and the capillary temperature was 300 °C. High-purity argon gas (purity ≥ 99.999%) was used as the collision gas. Nitrogen gas (purity ≥ 99.999%) was used as the sheath gas at 40 Arb and as the auxiliary gas at 15 Arb, with an auxiliary gas heater temperature of 350 °C. The total data acquisition time was 30.0 min.

Chromatographic separation was performed on an AQ-C18 column (150 × 2.1 mm, 1.8 μm, Welch). The flow rate was set at 0.30 mL/min. The mobile phase consisted of 0.1% formic acid in water (aqueous phase) and methanol (organic phase). The column oven temperature was maintained at 35 °C, and the autosampler temperature was set at 10.0 ℃. The injection volume was 5.00 μL. The gradient elution program is shown in Table 7.

### 3.9. In Vitro Antioxidant Activity Study of CDEs

#### 3.9.1. Evaluation of DPPH Radical Scavenging Ability

According to the method of Brand-Williams et al. [42], 500 μL of extract solutions at various concentrations (0.0625 mg/mL, 0.125 mg/mL, 0.25 mg/mL, 0.5 mg/mL, and 1 mg/mL) and 500 μL of vitamin C (VC) solutions at various concentrations (0.625 μg/mL, 1.25 μg/mL, 2.5 μg/mL, 5 μg/mL, and 10 μg/mL) were each mixed with 500 μL of DPPH solution. The mixtures were thoroughly blended and incubated in the dark at room temperature for 30 min. Absorbance at 517 nm (A_517_) was measured, with each sample tested in triplicate. Vitamin C solution was used as a positive control, and 50% ethanol was used as a blank control. The DPPH radical scavenging rate at each concentration was calculated according to Equation (3):

DPPH Scavenging Rate (%) = (1 − A_1_/A_0_) × 100% (3)

Note: A_1_ is the absorbance at 517 nm of the sample group; A_0_ is the absorbance at 517 nm of the blank control group.

#### 3.9.2. Evaluation of ABTS Radical Scavenging Activity

According to the method described by Re et al. [43], 500 μL of extract solutions at various concentrations (0.0625 mg/mL, 0.125 mg/mL, 0.25 mg/mL, 0.5 mg/mL, 1 mg/mL) and 500 μL of vitamin C (VC) solutions at different concentrations (0.625 μg/mL, 1.25 μg/mL, 2.5 μg/mL, 5 μg/mL, 10 μg/mL) were mixed with 500 μL of ABTS solution. The mixtures were thoroughly mixed and incubated at room temperature for 30 min in the dark. Absorbance was measured at 734 nm (A_734_), with each sample analyzed in triplicate. Vitamin C was used as the positive control, and 50% ethanol served as the blank control. The ABTS radical scavenging rate at each concentration was calculated using Equation (4):

ABTS Scavenging Rate (%) = (1 − A_1_/A_0_) × 100 (4)

Note: A_1_ is the absorbance at 734 nm of the sample group; A_0_ is the absorbance at 734 nm of the blank control group.

### 3.10. In Vitro Antibacterial Activity of CDE

The MIC of CDE was measured using the broth microdilution method, following Clinical and Laboratory Standards Institute (CLSI) guidelines. A 96-well microplate was prepared with 100 μL of Mueller–Hinton (MH) broth in each well. 100 μL of CDE at an initial concentration of 1024 mg/mL was added to the first well of the first row. Two-fold serial dilutions were performed to obtain 10 concentration gradients: 512, 256, 128, 64, 32, 16, 8, 4, 2, and 1 mg/mL. Subsequently, 100 μL of bacterial suspension was added to each well. Well 11, containing MH broth and bacterial suspension but no extract, served as the positive control, while well 12, containing only MH broth, served as the negative control. The plate was incubated at 37 °C for 24 h, and bacterial growth in each well was observed to determine the MIC of the extract against each bacterial strain.

### 3.11. Statistical Analysis

RSM experimental data were statistically analyzed using Design Expert 8.0.6 software (Trial Version 8.0.6, State, Inc., Minneapolis, MN, USA). Other data were analyzed with SPSS 17.0 software (SPSS Inc., Chicago, IL, USA). IC_50_ values were calculated using GraphPad Prism 8.0 (GraphPad Software, San Diego, CA, USA) by nonlinear regression. The radical scavenging rates were plotted against the logarithm of extract concentrations. The concentration required to achieve 50% scavenging (IC_50_) was derived from the fitted dose–response curve. All data are expressed as mean ± standard deviation (SD). A value of *p* < 0.05 was considered statistically significant. High-resolution LC-MS data were initially processed using Compound Discoverer 3.3 (Thermo Fisher, Waltham, MA, USA) and then matched against the mzCloud database.

## 4. Conclusions

In this study, an ultrasound-assisted ethanol extraction method for Cortex Dictamni was optimized using response surface methodology. The optimal conditions were: ethanol concentration of 70%, extraction time of 35 min, and material-to-liquid ratio of 1:40 g/mL. The resulting crude extract (CDE) contained alkaloids, flavonoids, terpenoids, and lactones as identified by LC-MS. CDE exhibited moderate DPPH and ABTS radical scavenging activities (IC_50_ = 0.316 and 0.166 mg/mL, respectively) and weak antibacterial activity against *E. coli* and *P. aeruginosa* (MIC = 128 mg/mL). These findings suggest that CDE has potential as a natural antioxidant, but further purification and mechanistic studies are required to fully evaluate its antibacterial potential.

## Figures and Tables

**Table 1:** Box–Behnken design with experimental factors and the observed extraction yield of CDE.

No.	*A* Material-Liquid Ratio/(mL/g)	*B* Ethanol Concentration/%	*C* Extraction Time/Min	*Y* Extraction Rate/%
1	−1	0	1	22.32
2	−1	1	0	19.26
3	0	0	0	22.72
4	1	−1	0	21.78
5	1	0	1	21.96
6	0	0	0	46.08
7	0	0	0	54.18
8	1	1	0	25.02
9	0	1	1	16.38
10	0	1	−1	19.62
11	−1	0	−1	18.18
12	0	0	0	40.86
13	−1	−1	0	24.66
14	0	−1	−1	20.52
15	0	0	0	44.1
16	0	−1	1	22.86
17	1	0	−1	14.76

**Table 2:** Analysis of variance.

Source	Sum of Squares	df	Mean Square	*F*-Value	*p*-Value
Model	7.99	9	0.8876	14.10	0.0011
A	0.0003	1	0.0003	0.0050	0.9458
B	0.0351	1	0.0351	0.5577	0.4795
C	0.0420	1	0.0420	0.6678	0.4407
AB	0.0576	1	0.0576	0.9148	0.3707
AC	0.0072	1	0.0072	0.1147	0.7447
BC	0.0240	1	0.0240	0.3816	0.5563
A^2^	2.05	1	2.05	32.58	0.0007
B^2^	1.88	1	1.88	29.84	0.0009
C^2^	3.08	1	3.08	48.94	0.0002
Residual	0.4407	7	0.0630		
Lack of Fit	0.0854	3	0.0285	0.3206	0.8115
Pure Error	0.3553	4	0.0888		
Cor Total	8.43	16			
R^2^	0.9477				
R^2^_Adj_	0.8805				

Note: *p* < 0.01 indicates a highly significant difference; *p* < 0.05 indicates a significant difference.

**Table 3:** Identification of chemical components in CDE by LC-MS.

No.	Rt (Min)	[M − H]^−^	MS/MS [M − H]^−^	[M + H]^−^	MS/MS [M + H]^−^	Calculated Mass	Formula	Proposed Molecule	Reference
1	8.69	—	—	169.1	151.1 164.8 169.1	168.1	C11 H8 N2	Norharman	[18]
2	8.82	—	—	200.1	185.1 129.1 144.1	199.2	C12 H9 NO2	Dictamnine	[19]
3	15.91	—	—	260.1	199.1 227.1 260.1	259.1	C14 H13 N O4	Skimmianine	[20]
4	1.43	—	—	138.1	92.1 110.1 138.1	137.1	C7 H7 N O2	Trigonelline	[21]
5	12.83	—	—	609.2	151.0 257.1 301.1	550.2	C28 H34 O15	Neohesperidin	[22]
6	14.46	—	—	471.2	95.0 105.1 161.1	470.2	C26 H30 O8	Limonoids	[23]
7	17.91	—	—	459.1	401.1 427.1 459.1	458.1	C21 H24 O10	Phlorizin	[24]
8	16.84	—	—	455.2	95.1 105.1 161.1	454.2	C26 H30 O7	Obacunone	[25]
9	14.35	—	—	177.1	135.1 159.1 177.1	194.1	C12 H18 O2	Sedanolide	[26]
10	18.62	—	—	333.2	301.1 333.2	332.2	C20 H30 O5	Andrographolide	[27]

**Table 4:** The results of the antioxidant activities of CDE.

Indicators	Antioxidants	Equation of Equations	R^2^ of Linear Fit	IC_50_
DPPH	CDE	y = 61.196 x + 80.56	0.9918	0.316 (mg/mL)
	VC	y = 34.119 x + 62.641	0.9921	1.25 (μg/mL)
ABTS+	CDE	y = 58.667 x + 95.9	0.9926	0.166 (mg/mL)
	VC	y = 51.367 x + 97.38	0.9934	0.43 (μg/mL)

**Table 5:** MIC of CDE from Cortex Dictamni.

Bacterial Strain	Concentration of Extracts (mg/mL)									
	512	256	128	64	32	16	8	4	2	1
*Staphyloccocus aureus*	+	+	+	+	+	+	+	+	+	+
*Escherichia coli*	-	-	-	+	+	+	+	+	+	+
*Salmonella enterica*	-	+	+	+	+	+	+	+	+	+
*listeria monocytogenes*	-	-	+	+	+	+	+	+	+	+
*Pseudomonas aeruginosa*	-	-	-	+	+	+	+	+	+	+

Note: “-” indicates no bacterial growth; “+” indicates bacterial growth.

**Table 6:** Independent variables and their levels in Box–Behnken design.

Factors	Levels		
	−1	0	1
A material-liquid ratio/(g/mL)	1:30	1:40	1:50
B ethanol concentration/%	55	65	75
C extraction time/min	30	40	50

**Table 7:** Chromatographic gradient elution.

Time (Min)	Water Phase Ratio (%)	Organic Phase Ratio (%)
1	98	2
5	80	20
10	50	50
15	20	80
20	5	95
27	5	95
28	98	2
30	98	2

## Data Availability

The original contributions presented in the study are included in the article; further inquiries can be directed to the corresponding authors.
