# Correction: Chen et al. Optimization of Extraction, Chemical Profiling, and Evaluation of Antioxidant and Antibacterial Activities of Cortex Dictamni Extract. *Molecules* 2026, *31*, 1869

**DOI:** 10.3390/molecules31142458

**Published:** 2026-07-14

**Authors:** Hui Chen, Mingfang Teng, Hang Zhao, Huining Zhang, Meihui Yang, Peng Zhang, Rongfeng Chen

**Affiliations:** 1 National Medical Products Administration Institute of Executive Development, Beijing 100073, China; 2 College of Life Engineering, Shenyang Institute of Technology, Fushun 113122, China; 3 National Center for Occupational Safety and Health, National Health Commission, Beijing 102308, China

In the original publication [1], there was a mistake in the text of Section 2.4. Identification of Chemical Components in CDE, as published. The text “Of the ten compounds identified, norharman, skimmianine, and trigonelline are alkaloids; hesperidin, limonoids, and dictamnine glycoside are terpenoids; serdanolide and andrographolide are lactones; and the remaining two compounds are flavonoids.” appeared wrongly.

The corrected version of text appears below:

“Of the ten compounds identified, norharman, dictamnine, skimmianine, and trigonelline are alkaloids; limonin and obacunone are terpenoids; sedanolide and andrographolide are lactones; and phlorizin and neohesperidin are flavonoids.”

The authors state that the scientific conclusions are unaffected. This correction was approved by the Academic Editor. The original publication has also been updated.
